# Supplementary material for: A fourth-generation high-dimensional neural network potential with accurate electrostatics including non-local charge transfer
Source: Nat Commun. 2021 Jan 15;12:398. doi: 10.1038/s41467-020-20427-2 (PMC7811002; doi:10.1038/s41467-020-20427-2)
Supplement: Supplementary file 1 — Supplementary Information [file 41467_2020_20427_MOESM1_ESM.pdf]

Supplementary Information

A Fourth-Generation High-Dimensional Neural Network Potential with Accurate Electrostatics Including Non-local Charge Transfer

Tsz Wai Ko, Jonas A Finkler, Stefan Goedecker, Jörg Behler

December 26, 2020

Contents

Supplementary Methods **2**
Overview 2
Charge equilibration 2
Derivatives used for the calculation of the forces and for the neural network training 3
Calculation of $\frac{dQ_i}{dr_\alpha}$ 4
Calculation of $\frac{dQ_i}{d\chi_i}$ and $\frac{dQ_i}{dJ_i}$ 4
An efficient method for the force computation 4
Charge equilibration for periodic systems 5
Calculation of $\frac{dA_{ij}}{dr_\alpha}$ 6
Short-range part 7

Supplementary Tables **8**
$C_{10}H_2/C_{10}H_3^+$ 8
$Ag_3^{+/-}$ clusters 11
$Na_{8/9}Cl_8^+$ clusters 13
Au_2-MgO 16

Supplementary Figures **19**

Supplementary References **24**

Supplementary Methods

Overview

Here, we provide the technical details of the 4G-HDNNP method. The total energy consists of two parts, the short-range energy and long-range electrostatic energy,

$$E_{\text{total}} = E_{\text{elec}} + E_{\text{short}} \quad . \tag{1}$$

The electrostatic energy is calculated from the atomic charges, which are obtained from a charge equilibration scheme (Qeq) based on environment-dependent electronegativities that are predicted by atomic neural networks. The short-range energy is a sum of atomic energies computed by atomic neural networks, which compared to 2D-HDNNPs have an additional input node providing the atomic charge. With this additional information the short-range atomic neural networks are able to accurately predict energetic changes due to modifications in the local electronic structure resulting from long-range charge transfer. Atomic forces are calculated by taking the negative analytic derivatives of the total energy in Eq. 1 with respect to the atomic positions.

Charge equilibration

The charge equilibration method [1] is based on the idea that the electrons in a system are distributed in a way that minimizes the total energy. This energy does not only include the electrostatic Coulomb terms but also a term that describes the local energy caused by some amount of charge on an atom. This local energy is usually described using a Taylor series up to second order. The expansion factor for the linear term is called the electronegativity (χ_i), while the second order factor is called the atomic hardness (J_i). The charge on each atom is Gaussian distributed with width σ_i. This results in the energy expression for the charge equilibration scheme

$$E_{\text{Qeq}} = E_{\text{elec}} + \sum_{i=1}^{N_{\text{at}}} \chi_i Q_i + \frac{1}{2} J_i Q_i^2 \quad . \tag{2}$$

N_{at} is the number of atoms in the system. E_{elec} is the electrostatic energy resulting from the Gaussian charge distributions,

$$E_{\text{elec}} = \sum_{\text{i<j}}^{N_{\text{at}}} \frac{\text{erf}\left(\frac{r_{ij}}{\sqrt{2}\gamma_{ij}}\right)}{r_{ij}} Q_i Q_i + \sum_{i=1}^{N_{\text{at}}} \frac{Q_i^2}{2\sigma_i\sqrt{\pi}} \tag{3}$$

and

$$\gamma_{ij} = \sqrt{\sigma_i^2 + \sigma_j^2} \quad . \tag{4}$$

As only linear and quadratic terms in Q_i appear in E_{elec} and E_{Qeq}, they can be expressed using matrix notation

$$[\mathbf{E}]_{ij} = \begin{cases} \frac{1}{\sigma_i\sqrt{\pi}}, & \text{if } i = j \\ \frac{\text{erf}\left(\frac{r_{ij}}{\sqrt{2}\gamma_{ij}}\right)}{r_{ij}}, & \text{otherwise} \end{cases} \tag{5}$$

$$E_{\text{elec}} = \frac{1}{2}\mathbf{Q}^\top \mathbf{E}\mathbf{Q} \tag{6}$$

and

$$E_{\text{Qeq}} = \frac{1}{2}\mathbf{Q}^\top \mathbf{A}\mathbf{Q} + \mathbf{Q}^\top \boldsymbol{\chi} \quad , \tag{7}$$

with $\mathbf{Q}$ being a column vector containing the atomic charges Q_i, $\boldsymbol{\chi}$ being a column vector of the electronegativities and $\mathbf{A}$ being the matrix

$$[\mathbf{A}]_{ij} = \begin{cases} J_i + \frac{1}{\sigma_i\sqrt{\pi}}, & \text{if } i = j \\ \frac{\mathrm{erf}\left(\frac{r_{ij}}{\sqrt{2}\gamma_{ij}}\right)}{r_{ij}}, & \text{otherwise} \end{cases} \quad . \tag{8}$$

It has to be noted that because the total energy of a continuous charge distribution is always positive, the matrix $\mathbf{A}$ is positive definite, if the $J_i > 0$.

The Q_i are now chosen, so that they minimize the energy E_{Qeq} under the additional constraint of total charge conservation

$$\sum_{i=1}^{N_{\mathrm{at}}} Q_i = Q_{\mathrm{tot}} \quad . \tag{9}$$

To solve this minimization problem, we set the derivatives with respect to the charges to zero,

$$\frac{dE_{\mathrm{Qeq}}}{dQ_i} = 0 \quad . \tag{10}$$

Including the constraint of total charge conservation using a Lagrange multiplier λ we end up with the system of linear equations

$$\left(\begin{array}{ccc|c} & & & 1 \\ & \mathbf{A} & & \vdots \\ & & & 1 \\ \hline 1 & \dots & 1 & 0 \end{array}\right) \left(\begin{array}{c} Q_1 \\ \vdots \\ Q_{N_{\mathrm{at}}} \\ \hline \lambda \end{array}\right) = \left(\begin{array}{c} -\chi_1 \\ \vdots \\ -\chi_{N_{\mathrm{at}}} \\ \hline Q_{\mathrm{tot}} \end{array}\right) \quad , \tag{11}$$

which we can rewrite for simplicity as

$$\mathbf{A}'\mathbf{Q}' = \mathbf{b} \quad , \tag{12}$$

where $\mathbf{A}'$ and $\mathbf{Q}'$ represent the $(N_{\mathrm{at}} + 1 \times N_{\mathrm{at}} + 1)$ matrix and column vector in the left hand side respectively, while $\mathbf{b}$ is the column vector on the right hand side. The electronegativities χ_i are predicted by neural networks, for each atom individually, depending on the local chemical environments. The hardness values of J_i are constant for a given element, and they are also optimized during the training of the neural networks.

Derivatives used for the calculation of the forces and for the neural network training

In this section we provide further details about some of the derivatives which are required for the calculation of the atomic forces as well as for the gradient-based optimization of the neural network parameters. The atomic force component F_{r_α} is given as the negative derivative of the energy with respect to the atomic coordinate r_α,

$$F_{r_\alpha} = -\frac{dE_{\mathrm{total}}(\mathbf{R}, \mathbf{Q}(\mathbf{R}))}{dr_\alpha} = \frac{\partial E_{\mathrm{total}}}{\partial r_\alpha} - \sum_i \frac{\partial E_{\mathrm{total}}}{\partial Q_i}\frac{\partial Q_i}{\partial r_\alpha} \quad . \tag{13}$$

In this equation the partial derivatives of the atomic charges with respect to the atomic positions appear. As we will show in section *An efficient method for the force computation*, the calculation of these terms can be avoided for the determination of the forces. During the training phase, however, these derivatives as well as the derivatives of the charges w.r.t. the electronegativity and hardness are needed.

Calculation of $\frac{dQ_i}{dr_\alpha}$

To calculate the $\frac{\partial Q_i}{\partial r_\alpha}$ we take the derivative with respect to the spatial coordinate r_α of the Qeq Eq. 12. Reordering the terms yields

$$\mathbf{A}\frac{\partial \mathbf{Q}}{\partial r_\alpha} = \frac{-\partial \boldsymbol{\chi}}{\partial r_\alpha} - \frac{\partial \mathbf{A}}{\partial r_\alpha}\mathbf{Q} \tag{14}$$

as well as a Lagrange multiplier that ensures $\sum_i \frac{\partial Q_i}{\partial r_\alpha} = 0$. To obtain all the $3N_{\mathrm{at}}$ required derivatives we will have to solve 3 N_{at} linear equation systems of size $N_{\mathrm{at}}+1$. However, this can be avoided as explained in section *An efficient method for the force computation*, which allows the calculation of the total force by only solving one linear equation system.

Calculation of $\frac{dQ_i}{d\chi_i}$ and $\frac{dQ_i}{dJ_i}$

With an similar procedure we can calculate the equation systems for the derivatives w.r.t. the electronegativity and hardness

$$\mathbf{A}\frac{d\mathbf{Q}}{d\chi_i} = -\boldsymbol{\delta}_i \tag{15}$$

and

$$\mathbf{A}\frac{d\mathbf{Q}}{dJ_i} = -\mathbf{Q} \quad . \tag{16}$$

$\boldsymbol{\delta}_i$ is a vector filled with zeros except entry i, which is one. As before, a Lagrange multiplier will be necessary, to ensure that the sum of the derivatives adds up to zero.

An efficient method for the force computation

In the last section we showed how the forces can be calculated using the partial derivatives of the charges w.r.t. the atomic coordinates. This calculation is computationally expensive, since 3 N_{at} linear equation systems need to be solved. This can be avoided by exploiting a method [2] that allows the calculation of the forces by solving only one linear equation system instead.

The total energy of the 4G-HDNNP is a function of the atomic coordinates ($\mathbf{R}$) and the charges ($\mathbf{Q}$), which also depend on the atomic coordinates,

$$E_{\mathrm{total}} = E_{\mathrm{total}}(\mathbf{R}, \mathbf{Q}(\mathbf{R})) \quad . \tag{17}$$

We now define an auxiliary function L with

$$L = E_{\mathrm{total}} + \sum_{i=1}^{N_{\mathrm{at}}+1} \lambda_i \left(\sum_{j=1}^{N_{\mathrm{at}}+1} A'_{ij} Q'_j - b_i \right) \quad . \tag{18}$$

Here $\sum_{j=1}^{N_{\mathrm{at}}+1} A'_{ij} Q'_j - b_i$ are the differences of the left hand side minus the right hand sides of Eq. 12, which were solved to determine the charges Q_i. These terms are therefore always zero, making L equal to E_{total}. We now choose λ such that the partial derivatives $\frac{\partial L}{\partial Q'_i}$ are zero,

$$\frac{\partial L}{\partial Q'_i} = \frac{\partial E_{\mathrm{total}}}{\partial Q'_i} + \sum_{j=1}^{N_{\mathrm{at}}+1} A'_{ij}\lambda_j = 0 \quad . \tag{19}$$

For this we solve the linear equation system

$$\sum_{j=1}^{N_{\text{at}}+1} A'_{ij}\lambda_j = \frac{-\partial E_{\text{total}}}{\partial Q'_i} \quad . \tag{20}$$

Note that $\mathbf{A}'$ is a symmetric matrix. We now turn to the derivative $\frac{dL}{dr_\alpha}$, which is equal to $\frac{dE_{\text{total}}}{dr_\alpha}$.

$$\frac{dE_{\text{total}}}{dr_\alpha} = \frac{dL}{dr_\alpha} = \frac{\partial E_{\text{total}}}{\partial r_\alpha} + \sum_{i=1}^{N_{\text{at}}+1} \frac{\partial E_{\text{total}}}{\partial Q'_i}\frac{\partial Q'_i}{\partial r_\alpha} + \sum_{i=1}^{N_{\text{at}}+1} \lambda_i \left(\sum_{j=1}^{N_{\text{at}}+1} \frac{\partial A'_{ij}}{\partial r_\alpha} Q'_j + \sum_{j=1}^{N_{\text{at}}+1} A'_{ij} \frac{\partial Q'_j}{\partial r_\alpha} - \frac{\partial b_i}{\partial r_\alpha} \right) \tag{21}$$

Rearranging the equation yields

$$\frac{dE_{\text{total}}}{dr_\alpha} = \frac{dL}{dr_\alpha} = \frac{\partial E_{\text{total}}}{\partial r_\alpha} + \sum_{i=1}^{N_{\text{at}}+1} \left(\frac{\partial E_{\text{total}}}{\partial Q'_i} + \sum_{j=1}^{N_{\text{at}}+1} A'_{ij}\lambda_j \right) \frac{\partial Q'_i}{\partial r_\alpha} + \sum_{i=1}^{N_{\text{at}}+1} \lambda_i \left(\sum_{j=1}^{N_{\text{at}}+1} \frac{\partial A'_{ij}}{\partial r_\alpha} Q'_j - \frac{\partial b_i}{\partial r_\alpha} \right) \quad . \tag{22}$$

The term $\frac{\partial E_{\text{total}}}{\partial Q'_i} + \sum_j A'_{ij}\lambda_j$ is zero by definition of λ and can therefore be omitted, which leads to the expression

$$\frac{dE_{\text{total}}}{dr_\alpha} = \frac{\partial E_{\text{total}}}{\partial r_\alpha} + \sum_{i=1}^{N_{\text{at}}+1} \lambda_i \left(\sum_{j=1}^{N_{\text{at}}+1} \frac{\partial A'_{ij}}{\partial r_\alpha} Q'_j - \frac{\partial b_i}{\partial r_\alpha} \right) \quad . \tag{23}$$

Charge equilibration for periodic systems

The Qeq equations for periodic boundary conditions are essentially identical to the corresponding equations for free boundary conditions, and the main difference is the calculation of the matrix $\mathbf{A}$. Because of the periodic boundary conditions we have to resort to an Ewald summation [3] to calculate the electrostatic interaction energy.

The basic idea of Ewald summation is, that by placing Gaussian charges of the opposite sign on each of the point charges, the remaining electrostatic interaction becomes short-ranged. This short-ranged energy can then be calculated in real space (E_{real}). We then subtract the interaction energy of the auxiliary Gaussian charges again to obtain the desired total energy of the point charges. This interaction energy of the Gaussians can be efficiently calculated in reciprocal space, resulting in the energies E_{recip} and E_{self}. The electrostatic energy of N_{at} point charges can be hence calculated as

$$E_{\text{elec}} = E_{\text{real}} + E_{\text{recip}} + E_{\text{self}} \quad . \tag{24}$$

The real space part is given by

$$E_{\text{real}}^{\text{pc}} = \frac{1}{2} \sum_{i=1}^{N_{\text{at}}} \sum_{j\neq i}^{N_{\text{neig}}} Q_i Q_j \frac{\operatorname{erfc}\left(\frac{r_{ij}}{\sqrt{2}\eta}\right)}{r_{ij}} \tag{25}$$

Here, N_{neig} indicates, that the sum goes over all neighbouring atoms withing the real space cutoff radius r_{cut}. r_{ij} is the distance between atoms i and j. The reciprocal space part is

$$E_{\text{recip}}^{\text{pc}} = \frac{2\pi}{V} \sum_{\mathbf{k}\neq 0} \frac{\exp\left(\frac{-\eta^2|\mathbf{k}|^2}{2}\right)}{|\mathbf{k}|^2} |S(\mathbf{k})|^2 \tag{26}$$

with

$$S(\mathbf{k}) = \sum_{i=1}^{N_{\text{at}}} Q_i \exp(i\mathbf{k} \cdot \mathbf{r}_i) \tag{27}$$

V being the volume of the unit cell and the sum going over all reciprocal lattice points inside reciprocal space cutoff radius $r_{\text{cut}}^{\text{recip}}$. Finally, the self-interaction correction is

$$E_{\text{self}}^{\text{pc}} = -\sum_{i=1}^{N_{\text{at}}} \frac{Q_i^2}{\sqrt{2\pi}\eta} \tag{28}$$

In these equations η is the standard deviation of the Gaussian charges, which are placed on the point charges to remove the long-range interactions.

Since we use Gaussian charge distributions for the charge equilibration process, the following terms have to be added that account for the different interaction in the short-range part as well as for the self interaction of the Gaussian charges [4, 5, 6].

$$E_{\text{elec}}^{\text{Gauss}} = E_{\text{elec}}^{\text{pc}} - \frac{1}{2}\sum_{i=1}^{N_{\text{at}}} \sum_{j\neq i}^{N_{\text{neig}}} Q_i Q_j \frac{\text{erfc}\left(\frac{r_{ij}}{\sqrt{2}\gamma}\right)}{r_{ij}} + \sum_{i=1}^{N_{\text{at}}} \frac{Q_i^2}{2\sqrt{\pi}\sigma_i} \tag{29}$$

Here $E_{\text{elec}}^{\text{pc}}$ is the electrostatic energy of the point charges as given above.

The important observation is that the total energy expression of the Ewald summation contains only terms of the form $\frac{1}{2}e_{ij}Q_iQ_j$. By calculating the individual coefficients e_{ij} we can therefore construct the matrix $\mathbf{E}$, so that

$$E_{\text{elec}} = \frac{1}{2}\mathbf{Q}^{\top}\mathbf{E}\mathbf{Q} \quad . \tag{30}$$

Including the terms for the hardness and adding the electronegativity results in a formalism equivalent to that of the Qeq method for free boundary condition.

Calculation of $\frac{dA_{ij}}{dr_\alpha}$

The differentiation of the above equation allows us to calculate the derivatives $\frac{dA_{ij}}{dr_\alpha}$. Explicit calculation of these derivatives however can be computationally expensive, as they are quite numerous ($3N_{\text{at}}^3$). Most of these coefficients however are zero, and the matrix is very sparse. As only the product $\sum_{j=1} \frac{dA_{ij}}{dr_\alpha} Q_j$ is ever needed in our computations, explicit calculation can be avoided and only the non zero terms have to be considered.

As $\sum_{j=1} \frac{dA_{ij}}{dr_\alpha} Q_j$ has to be calculated anyways for the efficient compuations of the forces, we can also use it to calculate the electrostatic forces.

$$\frac{\partial E_{\text{elec}}}{\partial r_\alpha} = \frac{1}{2}\sum_i Q_i \left(\sum_j \frac{dA_{ij}}{dr_\alpha} Q_j\right) \tag{31}$$

Calculation of the electrostatic energies and forces boils down to the following.

1. Calculate symmetry functions

2. Calculate environment depend electronegativities using a first set of neural networks

3. Construct the matrix $\mathbf{A}$

4. Calculate partial charges Q_i by solving the system of $N_{\text{at}}+1$ linear equations (Eq. 11)
5. Feed the atomic charges into the atomic NNs to calculate the short range energy and forces
6. Use the efficient method (section *An efficient method for the force computation*) to calculate the total force by solving one more $N_{\text{at}}+1$ dimensional linear equation system.

The only difference for the periodic case is in the step 3 and 6, where Ewald summation has to be used.

Short-range part

In the short range neural network, we also include the atomic charge via an additional input neuron, such that the atomic energy contribution also depends on global charge distributions. The expression of atomic energies is very similar to the 2G-HDNNP and it can be expressed as a function of symmetry functions and atomic charges. The atomic forces can be calculated by taking the derivatives of the energy with respect to the atomic positions

$$F_\alpha = -\sum_{i=1}^{N_{\text{at}}} \frac{dE_i}{dr_\alpha} = -\left(\sum_{j=1}^{N_{\text{neig},i}} \sum_{k=1}^{N_{\text{SF},j}} \frac{\partial E_j}{\partial G_{j,k}} \cdot \frac{\partial G_{j,k}}{\partial r_\alpha} + \sum_{j=1}^{N_{\text{at}}} \sum_{k=1}^{N_{\text{at}}} \frac{\partial E_j}{\partial Q_k} \cdot \frac{\partial Q_k}{\partial r_\alpha} \right) \tag{32}$$

where F_α and $G_{j,k}$ represent the force component α and the kth symmetry function of atom j respectively. In addition, $N_{\text{SF},i}$, $N_{\text{neig},i}$ equal to number of symmetry functions and neighbors of atom i and N_{at} the total number of atom. Note that $N_{\text{neig},i}$ includes the atom i itself. If the method described in section *An efficient method for the force computation* is used, the last term, which includes the partial derivatives of the atomic charges w.r.t. the atomic coordinates, can be avoided.

Supplementary Tables

$C_{10}H_2/C_{10}H_3^+$

Supplementary Table 1: Root mean square errors (RMSE) of charges (me), energies (meV/atom) and forces (meV/Å) for the three different HDNNP generations for the $C_{10}H_2/C_{10}H_3^+$ data set with 9035 and 984 structures for training and testing points respectively.

		charges	energy	forces
2G	train	—	1.583	130.7
	test	—	1.619	129.5
3G (unscaled)	train	27.36	3.192	652.5
	test	27.35	3.197	658.3
3G (scaled)	train	19.98	2.017	229.9
	test	20.08	2.045	231.0
4G	train	5.783	1.148	77.65
	test	6.577	1.194	78.00

Supplementary Table 2: Symmetry functions for $C_{10}H_2/C_{10}H_3^+$

no.	type	atom i	atom j	atom k	η(1/Bohr^2)	λ	ζ	R_c(Bohr)	R_s(Bohr)
1	2	H	H		0.0			8.0	0.0
2	2	H	H		0.006			8.0	0.0
3	2	H	H		0.011			8.0	0.0
4	2	H	H		0.018			8.0	0.0
5	2	H	H		0.026			8.0	0.0
6	2	H	H		0.035			8.0	0.0
7	2	C	H		0.0			8.0	0.0
8	2	C	H		0.013			8.0	0.0
9	2	C	H		0.029			8.0	0.0
10	2	C	H		0.054			8.0	0.0
11	2	C	H		0.093			8.0	0.0
12	2	C	H		0.161			8.0	0.0
13	2	H	C		0.0			8.0	0.0
14	2	H	C		0.013			8.0	0.0
15	2	H	C		0.029			8.0	0.0
16	2	H	C		0.054			8.0	0.0
17	2	H	C		0.093			8.0	0.0
18	2	H	C		0.161			8.0	0.0
19	2	C	C		0.0			8.0	0.0
20	2	C	C		0.01			8.0	0.0
21	2	C	C		0.023			8.0	0.0
22	2	C	C		0.041			8.0	0.0

no.	type	atom i	atom j	atom k	η(1/Bohr^2)	λ	ζ	R$_c$(Bohr)	R$_s$(Bohr)
23	2	C	C		0.065			8.0	0.0
24	2	C	C		0.103			8.0	0.0
25	3	C	C	C	0.0	1.0	1.0	8.0	
26	3	C	C	C	0.0	1.0	2.0	8.0	
27	3	C	C	C	0.0	1.0	4.0	8.0	
28	3	C	C	C	0.0	1.0	8.0	8.0	
29	3	C	C	C	0.0	-1.0	1.0	8.0	
30	3	C	C	C	0.0	-1.0	2.0	8.0	
31	3	C	C	C	0.0	-1.0	4.0	8.0	
32	3	C	C	C	0.0	-1.0	8.0	8.0	
33	3	C	H	H	0.0	1.0	1.0	8.0	
34	3	C	H	H	0.0	1.0	2.0	8.0	
35	3	C	H	H	0.0	1.0	4.0	8.0	
36	3	C	H	H	0.0	1.0	8.0	8.0	
37	3	C	H	H	0.0	-1.0	1.0	8.0	
38	3	C	H	H	0.0	-1.0	2.0	8.0	
39	3	C	H	H	0.0	-1.0	4.0	8.0	
40	3	C	H	H	0.0	-1.0	8.0	8.0	
41	3	C	C	H	0.0	1.0	1.0	8.0	
42	3	C	C	H	0.0	1.0	2.0	8.0	
43	3	C	C	H	0.0	1.0	4.0	8.0	
44	3	C	C	H	0.0	1.0	8.0	8.0	
45	3	C	C	H	0.0	-1.0	1.0	8.0	
46	3	C	C	H	0.0	-1.0	2.0	8.0	
47	3	C	C	H	0.0	-1.0	4.0	8.0	
48	3	C	C	H	0.0	-1.0	8.0	8.0	
49	3	H	C	C	0.0	1.0	1.0	8.0	
50	3	H	C	C	0.0	1.0	2.0	8.0	
51	3	H	C	C	0.0	1.0	4.0	8.0	
52	3	H	C	C	0.0	1.0	8.0	8.0	
53	3	H	C	C	0.0	-1.0	1.0	8.0	
54	3	H	C	C	0.0	-1.0	2.0	8.0	
55	3	H	H	C	0.0	1.0	1.0	8.0	
56	3	H	H	C	0.0	1.0	2.0	8.0	
57	3	H	H	C	0.0	1.0	4.0	8.0	
58	3	H	H	C	0.0	1.0	8.0	8.0	
59	3	H	H	C	0.0	-1.0	1.0	8.0	
60	3	H	H	C	0.0	-1.0	2.0	8.0	

Supplementary Table 3: DFT-optimized structure of $C_{10}H_2$ in XYZ format (distance unit in Å)

12 atoms

C	0.621 587 11	0.000 000 00	0.000 000 00
C	−0.621 587 10	0.000 000 00	0.000 000 00
C	1.952 478 40	0.000 000 00	0.000 000 00
C	−1.952 478 47	0.000 000 00	0.000 000 00
C	3.192 145 71	0.000 000 00	0.000 000 00
C	−3.192 145 71	0.000 000 00	0.000 000 00
C	4.535 930 62	0.000 000 00	0.000 000 00
C	−4.535 930 61	0.000 000 00	0.000 000 00
C	5.761 179 51	0.000 000 00	0.000 000 00
C	−5.761 179 49	0.000 000 00	0.000 000 00
H	6.831 800 63	0.000 000 00	0.000 000 00
H	−6.831 800 59	0.000 000 00	0.000 000 00

Supplementary Table 4: DFT-optimized structure of $C_{10}H_3^+$ in XYZ format (distance unit in Å)

13 atoms

C	0.629 569 25	0.000 007 77	0.000 008 66
C	−0.630 171 87	0.000 006 61	0.000 007 49
C	1.927 800 72	0.000 005 14	0.000 005 65
C	−1.939 451 25	0.000 003 66	0.000 004 44
C	3.196 698 75	−0.000 004 40	−0.000 004 75
C	−3.188 248 16	0.000 001 20	0.000 001 93
C	4.485 686 49	−0.000 026 21	−0.000 028 65
C	−4.518 203 60	−0.000 000 93	−0.000 000 22
C	5.792 415 11	−0.000 049 55	−0.000 059 76
C	−5.745 974 58	−0.000 002 54	−0.000 001 78
H	6.356 582 33	0.664 760 64	−0.662 349 90
H	−6.821 318 48	−0.000 003 46	−0.000 002 64
H	6.356 596 39	−0.664 669 86	0.662 408 63

$Ag_3^{+/-}$ clusters

Supplementary Table 5: Root mean square errors (RMSE) of charges (me), energies (meV/atom) and forces (meV/Å) for the three different HDNNP generations for the $Ag_3^{+/-}$ data set with 9930 and 1083 structures for training and testing points respectively.

		charges	energy	forces
2G	train	—	355.0	1812
	test	—	352.0	1803
3G (unscaled)	train	75.50	345.0	1909
	test	77.55	340.0	1963
3G (scaled)	train	26.24	321.1	1912
	test	26.48	320.2	1913
4G	train	10.61	1.293	32.12
	test	9.976	1.323	31.69

Supplementary Table 6: Symmetry functions for $Ag^{+/-}$ clusters

no.	type	atom i	atom j	atom k	η(1/Bohr^2)	λ	ζ	R_c(Bohr)	R_s(Bohr)
1	2	Ag	Ag		0.0			10.0	0.0
2	2	Ag	Ag		0.007			10.0	0.0
3	2	Ag	Ag		0.014			10.0	0.0
4	2	Ag	Ag		0.025			10.0	0.0
5	2	Ag	Ag		0.04			10.0	0.0
6	2	Ag	Ag		0.062			10.0	0.0
7	3	Ag	Ag	Ag	0.0	1.0	1.0	10.0	
8	3	Ag	Ag	Ag	0.0	1.0	2.0	10.0	
9	3	Ag	Ag	Ag	0.0	1.0	4.0	10.0	
10	3	Ag	Ag	Ag	0.0	1.0	8.0	10.0	
11	3	Ag	Ag	Ag	0.0	-1.0	1.0	10.0	
12	3	Ag	Ag	Ag	0.0	-1.0	2.0	10.0	

Supplementary Table 7: DFT-optimized structure of Ag_3^+ in XYZ format (distance unit in Å)

3 atoms

Ag	0.000 000 00	0.000 644 41	1.556 755 15
Ag	0.000 000 00	1.347 626 41	−0.779 404 15
Ag	0.000 000 00	−1.348 270 81	−0.777 351 99

Supplementary Table 8: DFT-optimized structure of Ag_3^- in XYZ format (distance unit in Å)

3 atoms

Ag	0.000 000 00	−1.326 870 06	2.317 857 81
Ag	0.000 000 00	1.340 265 98	−2.309 774 04
Ag	0.000 000 00	−0.013 395 92	−0.008 084 77

$Na_{8/9}Cl_8^+$ clusters

Supplementary Table 9: Root mean square errors (RMSE) of charges (me), energies (meV/atom) and forces (meV/Å) for the three different HDNNP generations for the $Na_{8/9}Cl_8^+$ data set with 4493 and 507 structures for training and testing points respectively.

		charges	energy	forces
2G	train	—	1.690	57.54
	test	—	1.692	57.39
3G (unscaled)	train	28.28	1.426	57.69
	test	28.52	1.470	59.49
3G (scaled)	train	20.75	2.058	73.47
	test	20.80	2.042	76.67
4G	train	15.87	0.474	32.45
	test	15.83	0.481	32.78

Supplementary Table 10: Symmetry functions for $Na_{8/9}Cl_8^+$ clusters

no.	type	atom i	atom j	atom k	η(1/Bohr^2)	λ	ζ	R$_c$(Bohr)	R$_s$(Bohr)
1	2	Na	Na		0.0			10.0	0.0
2	2	Na	Na		0.001			10.0	0.0
3	2	Na	Na		0.002			10.0	0.0
4	2	Na	Na		0.003			10.0	0.0
5	2	Na	Na		0.004			10.0	0.0
6	2	Na	Na		0.005			10.0	0.0
7	2	Na	Cl		0.0			10.0	0.0
8	2	Na	Cl		0.003			10.0	0.0
9	2	Na	Cl		0.005			10.0	0.0
10	2	Na	Cl		0.007			10.0	0.0
11	2	Na	Cl		0.01			10.0	0.0
12	2	Na	Cl		0.013			10.0	0.0
13	2	Cl	Na		0.0			10.0	0.0
14	2	Cl	Na		0.003			10.0	0.0
15	2	Cl	Na		0.005			10.0	0.0
16	2	Cl	Na		0.007			10.0	0.0
17	2	Cl	Na		0.01			10.0	0.0
18	2	Cl	Na		0.013			10.0	0.0
19	2	Cl	Cl		0.0			10.0	0.0
20	2	Cl	Cl		0.001			10.0	0.0
21	2	Cl	Cl		0.002			10.0	0.0
22	2	Cl	Cl		0.003			10.0	0.0
23	2	Cl	Cl		0.004			10.0	0.0
24	2	Cl	Cl		0.005			10.0	0.0

no.	type	atom i	atom j	atom k	$\eta(1/\text{Bohr}^2)$	λ	ζ	R_c(Bohr)	R_s(Bohr)
25	3	Na	Na	Cl	0.0	1.0	1.0	10.0	
26	3	Na	Na	Cl	0.0	1.0	2.0	10.0	
27	3	Na	Na	Cl	0.0	1.0	4.0	10.0	
28	3	Na	Na	Cl	0.0	1.0	8.0	10.0	
29	3	Na	Na	Cl	0.0	-1.0	1.0	10.0	
30	3	Na	Na	Cl	0.0	-1.0	2.0	10.0	
31	3	Na	Cl	Cl	0.0	1.0	1.0	10.0	
32	3	Na	Cl	Cl	0.0	1.0	2.0	10.0	
33	3	Na	Cl	Cl	0.0	-1.0	1.0	10.0	
34	3	Na	Cl	Cl	0.0	-1.0	2.0	10.0	
35	3	Na	Cl	Cl	0.0	-1.0	4.0	10.0	
36	3	Cl	Cl	Na	0.0	1.0	1.0	10.0	
37	3	Cl	Cl	Na	0.0	1.0	2.0	10.0	
38	3	Cl	Cl	Na	0.0	1.0	4.0	10.0	
39	3	Cl	Cl	Na	0.0	1.0	8.0	10.0	
40	3	Cl	Cl	Na	0.0	-1.0	1.0	10.0	
41	3	Cl	Na	Na	0.0	1.0	1.0	10.0	
42	3	Cl	Na	Na	0.0	1.0	2.0	10.0	
43	3	Cl	Na	Na	0.0	1.0	4.0	10.0	
44	3	Cl	Na	Na	0.0	-1.0	1.0	10.0	
45	3	Cl	Na	Na	0.0	-1.0	2.0	10.0	

Supplementary Table 11: DFT-optimized structure of $Na_9Cl_8^+$ in XYZ format (distance unit in Å)

17 atoms

Na	−4.672 483 91	1.744 323 61	0.066 917 32
Cl	−4.674 131 38	−0.763 117 13	−0.005 394 92
Cl	−2.156 850 54	2.353 451 40	0.126 851 59
Na	−2.120 583 99	−0.453 759 70	0.037 785 66
Na	0.430 089 05	2.067 507 92	0.061 237 48
Cl	0.514 739 28	−0.620 487 80	0.003 580 16
Cl	3.054 771 88	2.435 379 02	0.005 454 56
Na	3.081 187 44	−0.275 178 18	−0.020 881 71
Na	5.635 084 96	2.195 983 30	−0.008 956 06
Cl	5.728 609 48	−0.495 161 62	−0.005 581 27
Cl	8.304 179 09	2.518 938 87	0.008 721 19
Na	15.163 008 08	4.522 166 97	−0.019 004 50
Na	10.847 244 50	2.274 841 80	−0.037 879 82
Cl	10.992 739 88	−0.383 182 02	−0.002 413 13
Cl	13.644 077 08	2.525 223 09	0.039 396 59
Na	13.502 039 57	−0.152 682 89	0.114 915 06
Na	8.284 823 01	−0.197 007 56	0.036 113 70

Au_2–MgO

Supplementary Table 12: Root mean square errors (RMSE) of charges (me), energies (meV/atom) and forces (meV/Å) for the Au_2-MgO data set with 4468 and 532 structures for training and testing points respectively.

		charges	energy	forces
2G	train	—	2.299	155.4
	test	—	2.287	153.1
4G	train	5.663	0.209	81.05
	test	5.698	0.219	66.00

Supplementary Table 13: Symmetry functions for Au_2-MgO slabs

no.	type	atom i	atom j	atom k	η(1/Bohr^2)	λ	ζ	R_c(Bohr)	R_s(Bohr)
1	2	Mg	Mg		0.0			8.0	0.0
2	2	Mg	Mg		0.001			8.0	0.0
3	2	Mg	Mg		0.002			8.0	0.0
4	2	Mg	Mg		0.003			8.0	0.0
5	2	Mg	Mg		0.004			8.0	0.0
6	2	Mg	Mg		0.005			8.0	0.0
7	2	O	Mg		0.0			8.0	0.0
8	2	O	Mg		0.004			8.0	0.0
9	2	O	Mg		0.007			8.0	0.0
10	2	O	Mg		0.01			8.0	0.0
11	2	O	Mg		0.014			8.0	0.0
12	2	O	Mg		0.018			8.0	0.0
13	2	Mg	O		0.0			8.0	0.0
14	2	Mg	O		0.004			8.0	0.0
15	2	Mg	O		0.007			8.0	0.0
16	2	Mg	O		0.01			8.0	0.0
17	2	Mg	O		0.014			8.0	0.0
18	2	Mg	O		0.018			8.0	0.0
19	2	O	O		0.0			8.0	0.0
20	2	O	O		0.001			8.0	0.0
21	2	O	O		0.002			8.0	0.0
22	2	O	O		0.003			8.0	0.0
23	2	O	O		0.004			8.0	0.0
24	2	O	O		0.005			8.0	0.0
25	2	Mg	Au		0.0			8.0	0.0
26	2	Mg	Au		0.001			8.0	0.0
27	2	Mg	Au		0.002			8.0	0.0
28	2	Mg	Au		0.003			8.0	0.0

no.	type	atom i	atom j	atom k	$\eta(1/\mathrm{Bohr}^2)$	λ	ζ	R_c(Bohr)	R_s(Bohr)
29	2	Mg	Au		0.004			8.0	0.0
30	2	Mg	Au		0.005			8.0	0.0
31	2	Au	Mg		0.0			8.0	0.0
32	2	Au	Mg		0.001			8.0	0.0
33	2	Au	Mg		0.002			8.0	0.0
34	2	Au	Mg		0.003			8.0	0.0
35	2	Au	Mg		0.004			8.0	0.0
36	2	Au	Mg		0.005			8.0	0.0
37	2	Au	O		0.0			8.0	0.0
38	2	Au	O		0.004			8.0	0.0
39	2	Au	O		0.008			8.0	0.0
40	2	Au	O		0.013			8.0	0.0
41	2	Au	O		0.018			8.0	0.0
42	2	Au	O		0.024			8.0	0.0
43	2	O	Au		0.0			8.0	0.0
44	2	O	Au		0.004			8.0	0.0
45	2	O	Au		0.008			8.0	0.0
46	2	O	Au		0.013			8.0	0.0
47	2	O	Au		0.018			8.0	0.0
48	2	O	Au		0.024			8.0	0.0
49	2	Au	Au		0.0			8.0	0.0
50	2	Au	Au		0.004			8.0	0.0
51	2	Au	Au		0.008			8.0	0.0
52	2	Au	Au		0.012			8.0	0.0
53	2	Au	Au		0.017			8.0	0.0
54	2	Au	Au		0.022			8.0	0.0
55	2	O	Al		0.0			8.0	0.0
56	2	O	Al		0.003			8.0	0.0
57	2	O	Al		0.005			8.0	0.0
58	2	O	Al		0.008			8.0	0.0
59	2	O	Al		0.011			8.0	0.0
60	2	O	Al		0.014			8.0	0.0
61	2	Al	O		0.0			8.0	0.0
62	2	Al	O		0.003			8.0	0.0
63	2	Al	O		0.005			8.0	0.0
64	2	Al	O		0.008			8.0	0.0
65	2	Al	O		0.011			8.0	0.0
66	2	Al	O		0.014			8.0	0.0
67	2	Al	Mg		0.0			8.0	0.0
68	2	Al	Mg		0.001			8.0	0.0
69	2	Al	Mg		0.002			8.0	0.0
70	2	Al	Mg		0.003			8.0	0.0
71	2	Al	Mg		0.004			8.0	0.0
72	2	Al	Mg		0.005			8.0	0.0

no.	type	atom i	atom j	atom k	$\eta(1/\text{Bohr}^2)$	λ	ζ	R_c(Bohr)	R_s(Bohr)
73	2	Mg	Al		0.0			8.0	0.0
74	2	Mg	Al		0.001			8.0	0.0
75	2	Mg	Al		0.002			8.0	0.0
76	2	Mg	Al		0.003			8.0	0.0
77	2	Mg	Al		0.004			8.0	0.0
78	2	Mg	Al		0.005			8.0	0.0
79	3	Mg	Mg	Mg	0.0	1.0	1.0	8.0	
80	3	Mg	Mg	Mg	0.0	1.0	2.0	8.0	
81	3	Mg	Mg	Mg	0.0	1.0	4.0	8.0	
82	3	Mg	Mg	Mg	0.0	-1.0	1.0	8.0	
83	3	Mg	Mg	O	0.0	1.0	1.0	8.0	
84	3	Mg	Mg	O	0.0	1.0	2.0	8.0	
85	3	Mg	Mg	O	0.0	1.0	4.0	8.0	
86	3	Mg	Mg	O	0.0	1.0	8.0	8.0	
87	3	Mg	Mg	O	0.0	-1.0	1.0	8.0	
88	3	Mg	Mg	O	0.0	-1.0	2.0	8.0	
89	3	Mg	O	O	0.0	1.0	1.0	8.0	
90	3	Mg	O	O	0.0	1.0	2.0	8.0	
91	3	Mg	O	O	0.0	1.0	4.0	8.0	
92	3	Mg	O	O	0.0	-1.0	1.0	8.0	
93	3	Mg	O	O	0.0	-1.0	2.0	8.0	
94	3	Mg	O	O	0.0	-1.0	4.0	8.0	
95	3	Mg	O	Al	0.0	1.0	1.0	8.0	
96	3	Mg	O	Al	0.0	1.0	2.0	8.0	
97	3	Mg	O	Al	0.0	1.0	4.0	8.0	
98	3	Mg	O	Al	0.0	1.0	8.0	8.0	
99	3	Mg	O	Al	0.0	-1.0	1.0	8.0	
100	3	Mg	O	Au	0.0	1.0	1.0	8.0	
101	3	Mg	O	Au	0.0	1.0	2.0	8.0	
102	3	Mg	O	Au	0.0	1.0	4.0	8.0	
103	3	Mg	O	Au	0.0	1.0	8.0	8.0	
104	3	Mg	O	Au	0.0	-1.0	1.0	8.0	
105	3	Mg	O	Au	0.0	-1.0	2.0	8.0	
106	3	O	Mg	Mg	0.0	1.0	1.0	8.0	
107	3	O	Mg	Mg	0.0	1.0	2.0	8.0	
108	3	O	Mg	Mg	0.0	1.0	4.0	8.0	
109	3	O	Mg	Mg	0.0	-1.0	1.0	8.0	
110	3	O	Mg	Mg	0.0	-1.0	2.0	8.0	
111	3	O	Mg	Mg	0.0	-1.0	4.0	8.0	
112	3	O	Mg	O	0.0	1.0	1.0	8.0	
113	3	O	Mg	O	0.0	1.0	2.0	8.0	
114	3	O	Mg	O	0.0	1.0	4.0	8.0	
115	3	O	Mg	O	0.0	1.0	8.0	8.0	
116	3	O	Mg	O	0.0	-1.0	1.0	8.0	

no.	type	atom i	atom j	atom k	η(1/Bohr^2)	λ	ζ	R_c(Bohr)	R_s(Bohr)
117	3	O	Mg	O	0.0	-1.0	2.0	8.0	
118	3	O	Mg	Al	0.0	1.0	1.0	8.0	
119	3	O	Mg	Al	0.0	1.0	2.0	8.0	
120	3	O	Mg	Al	0.0	1.0	4.0	8.0	
121	3	O	Mg	Al	0.0	-1.0	1.0	8.0	
122	3	O	Mg	Al	0.0	-1.0	2.0	8.0	
123	3	O	Mg	Al	0.0	-1.0	4.0	8.0	
124	3	O	Mg	Au	0.0	1.0	1.0	8.0	
125	3	O	Mg	Au	0.0	1.0	2.0	8.0	
126	3	O	Mg	Au	0.0	-1.0	1.0	8.0	
127	3	O	Mg	Au	0.0	-1.0	2.0	8.0	
128	3	O	O	O	0.0	1.0	1.0	8.0	
129	3	O	O	O	0.0	1.0	2.0	8.0	
130	3	O	O	O	0.0	-1.0	1.0	8.0	
131	3	O	O	Al	0.0	1.0	1.0	8.0	
132	3	O	O	Al	0.0	1.0	2.0	8.0	
133	3	O	O	Al	0.0	-1.0	1.0	8.0	
134	3	O	O	Al	0.0	-1.0	2.0	8.0	
135	3	Al	Mg	Mg	0.0	1.0	1.0	8.0	
136	3	Al	Mg	O	0.0	1.0	1.0	8.0	
137	3	Al	Mg	O	0.0	1.0	2.0	8.0	
138	3	Al	Mg	O	0.0	-1.0	1.0	8.0	
139	3	Al	O	O	0.0	1.0	1.0	8.0	
140	3	Al	O	O	0.0	1.0	2.0	8.0	
141	3	Al	O	O	0.0	-1.0	1.0	8.0	
142	3	Al	O	O	0.0	-1.0	2.0	8.0	
143	3	Au	Mg	Mg	0.0	1.0	1.0	8.0	
144	3	Au	Mg	Mg	0.0	1.0	2.0	8.0	
145	3	Au	Mg	O	0.0	1.0	1.0	8.0	
146	3	Au	Mg	O	0.0	1.0	2.0	8.0	
147	3	Au	Mg	O	0.0	-1.0	1.0	8.0	
148	3	Au	Mg	O	0.0	-1.0	2.0	8.0	
149	3	Au	O	O	0.0	1.0	1.0	8.0	
150	3	Au	O	Au	0.0	1.0	-1.0	8.0	
151	3	Au	O	Au	0.0	1.0	-2.0	8.0	

Supplementary Figures

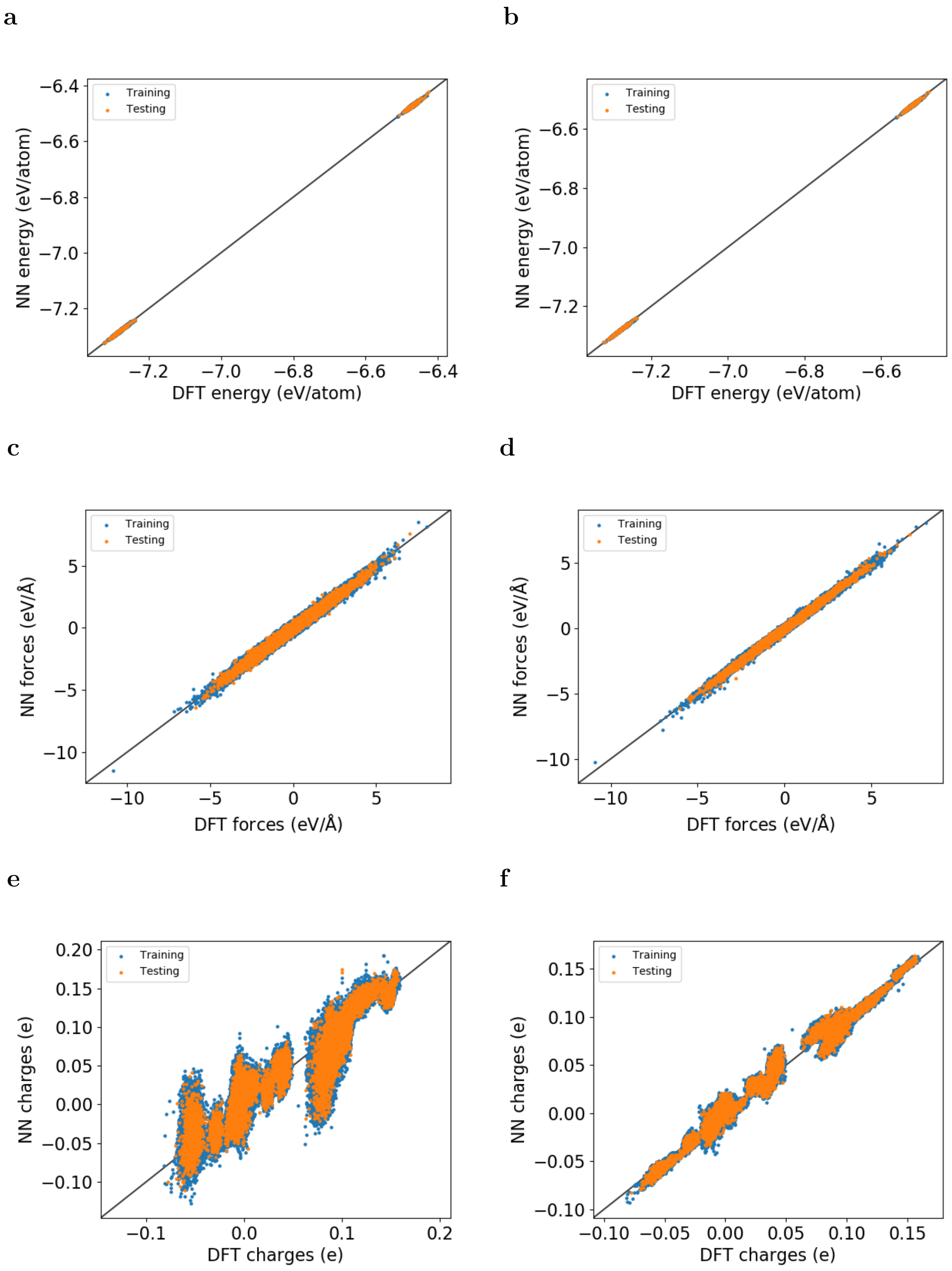

Supplementary Figure 1: Correlation plots of energies obtained from a 2G-HDNNP **a** and a 4G-HDNNP **b**, forces from a 2G-HDNNP **c** and a 4G-HDNNP **d** and charges from a scaled 3G-HDNNP **e** and a 4G-HDNNP **f** for $C_{10}H_2/C_{10}H_3^+$

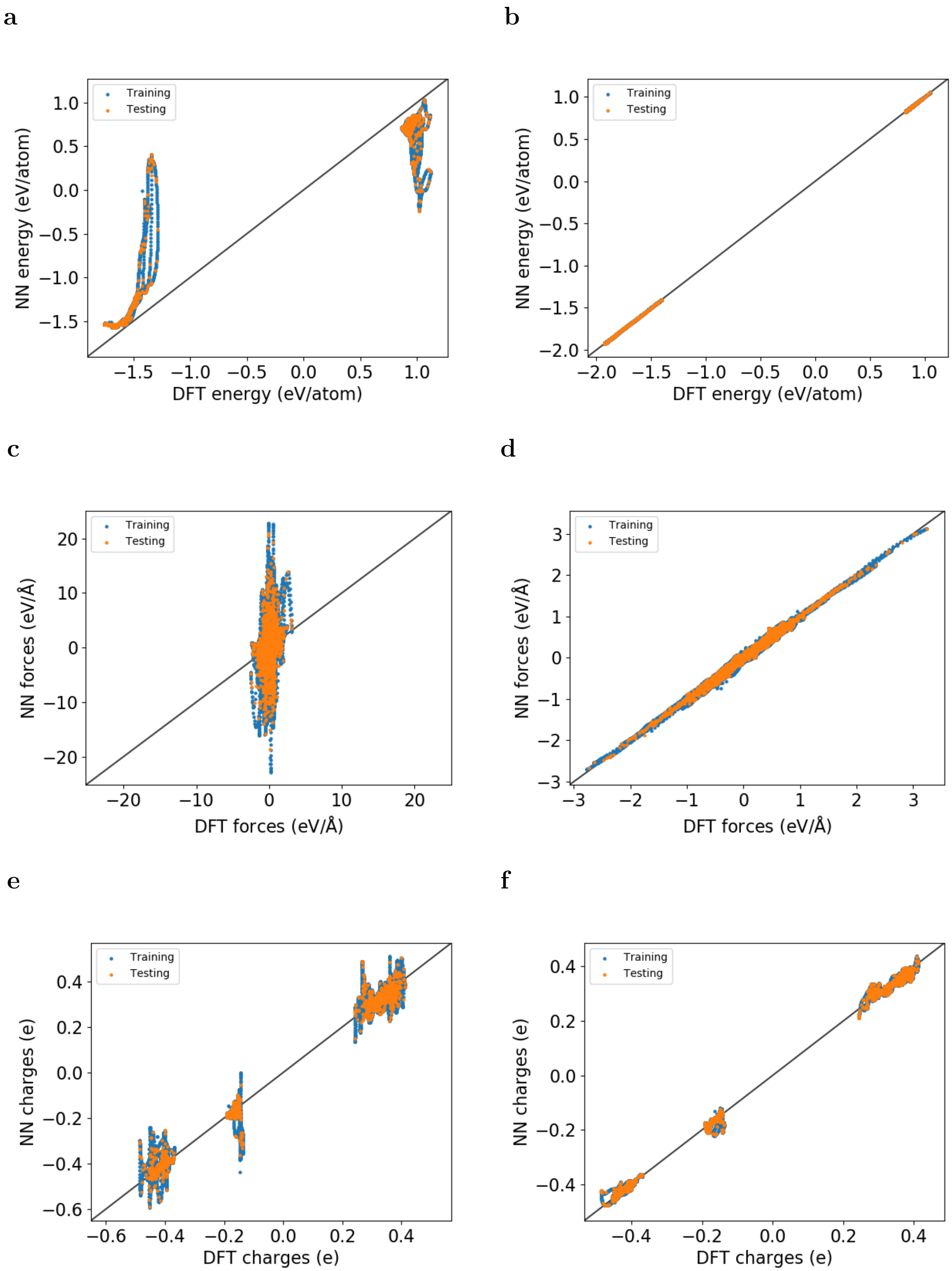

Supplementary Figure 2: Correlation plots of energies obtained from a 2G-HDNNP **a** and a 4G-HDNNP **b**, forces from a 2G-HDNNP **c** and a 4G-HDNNP **d** and charges from a scaled 3G-HDNNP **e** and a 4G-HDNNP **f** for $Ag_3^{+/-}$

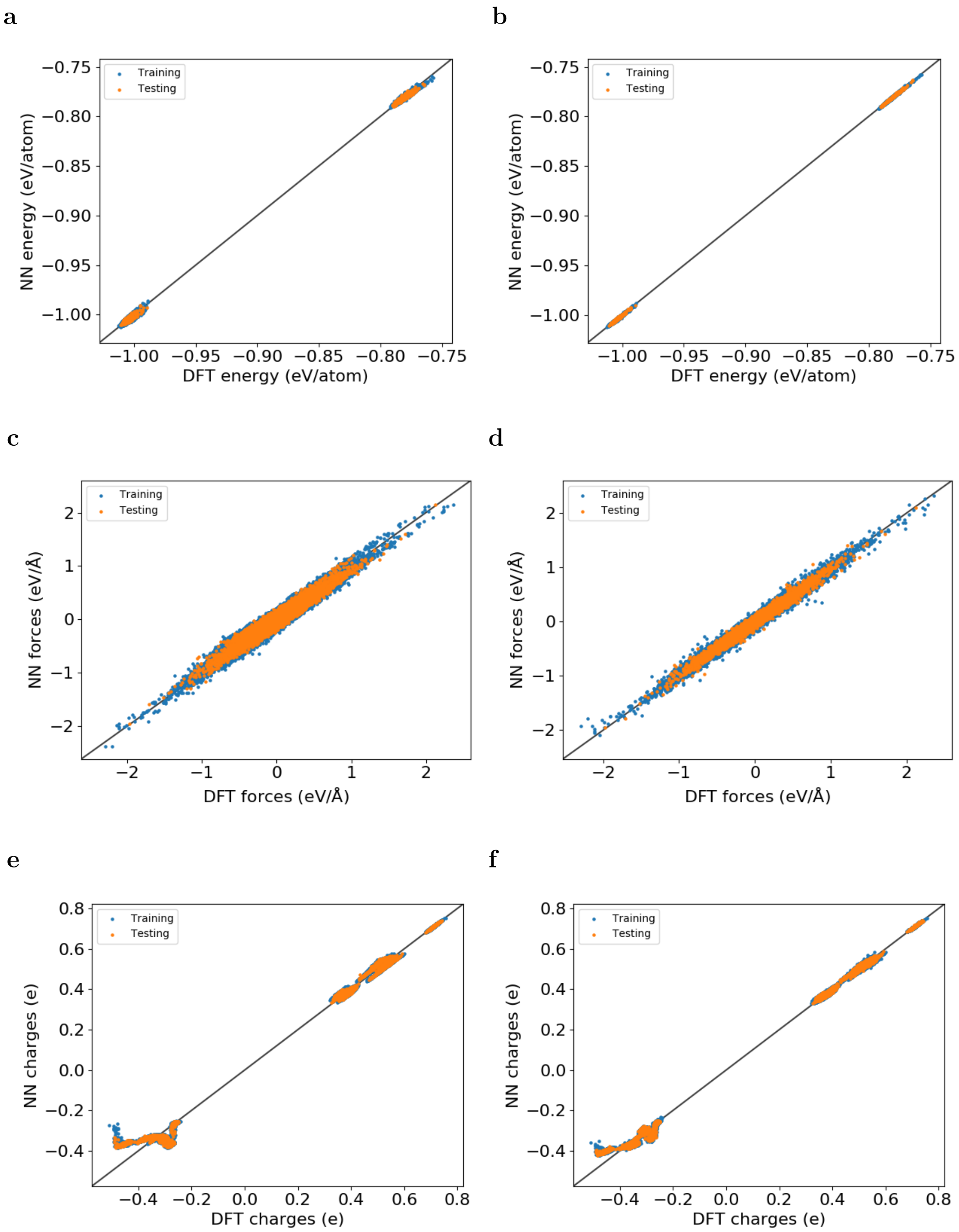

Supplementary Figure 3: Correlation plots of energies obtained from a 2G-HDNNP **a** and a 4G-HDNNP **b**, forces from a 2G-HDNNP **c** and a 4G-HDNNP **d** and charges from an unscaled 3G-HDNNP **e** and a 4G-HDNNP **f** for $Na_{8/9}Cl_8^+$

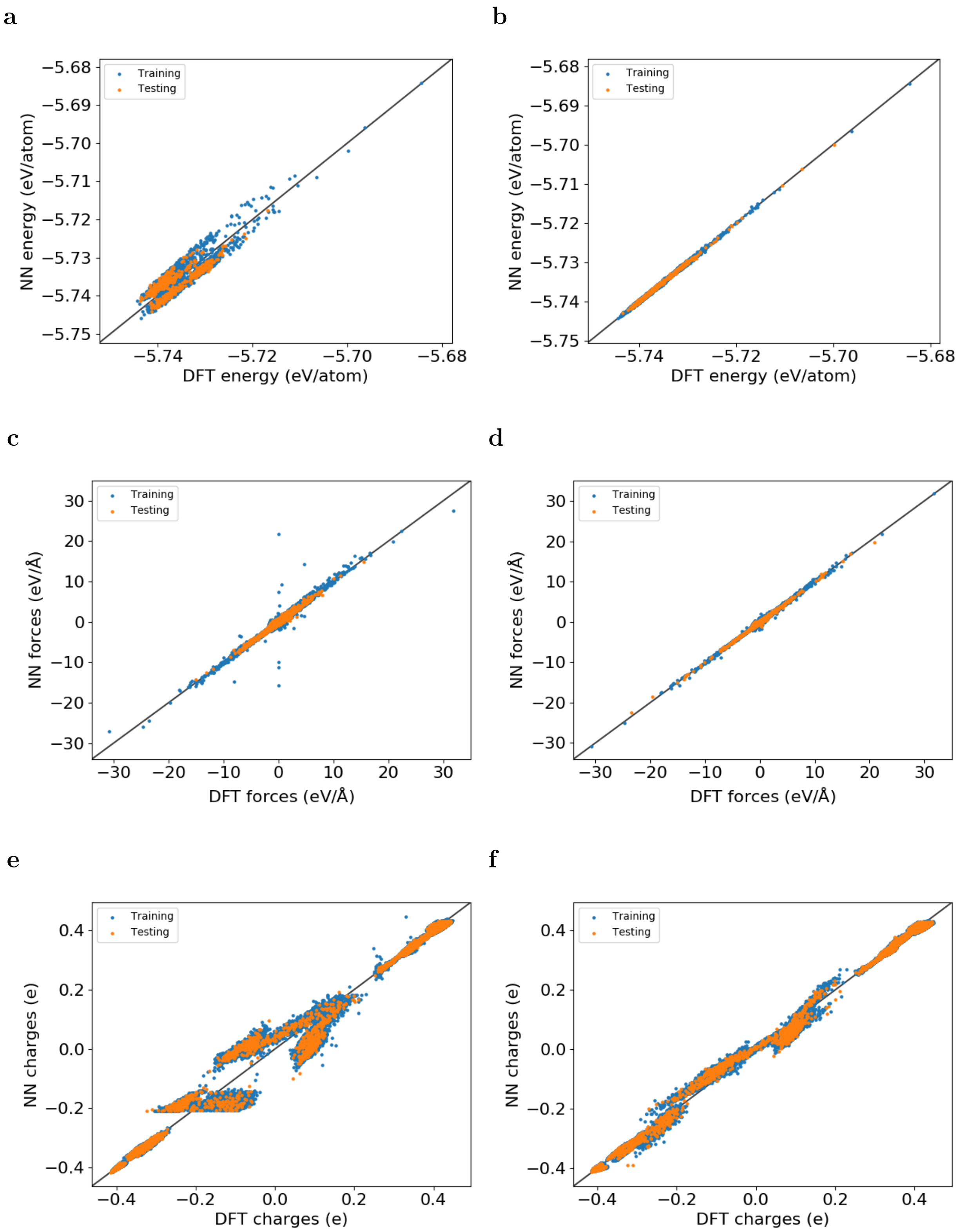

Supplementary Figure 4: Correlation plots of energies obtained from a 2G-HDNNP **a** and a 4G-HDNNP **b**, forces from a 2G-HDNNP **c** and a 4G-HDNNP **d** and charges from a scaled 3G-HDNNP **e** and a 4G-HDNNP **f** for Au_2-MgO

Supplementary References

[1] Rappe, A. K.; Goddard, W. A., III Charge equilibration for molecular dynamics simulations. *J. Phys. Chem.* **1991**, *95*, 3358.

[2] Poier, P. P.; Lagardère, L.; Piquemal, J.-P.; Jensen, F. Molecular Dynamics Using Nonvariational Polarizable Force Fields: Theory, Periodic Boundary Conditions Implementation, and Application to the Bond Capacity Model. *J. Chem. Theory Comput.* **2019**, *15*, 6213–6224.

[3] Ewald, P. P. Die Berechnung optischer und elektrostatischer Gitterpotentiale. *Annalen der physik* **1921**, *369*, 253–287.

[4] Gingrich, T. R.; Wilson, M. On the Ewald summation of Gaussian charges for the simulation of metallic surfaces. *Chem. Phys. Lett.* **2010**, *500*, 178–183.

[5] Kiss, P. T.; Sega, M.; Baranyai, A. Efficient handling of Gaussian charge distributions: An application to polarizable molecular models. *J. Chem. Theory Comput.* **2014**, *10*, 5513–5519.

[6] Lee, H.; Cai, W. Ewald summation for Coulomb interactions in a periodic supercell. *Lecture Notes, Stanford University* **2009**,
